# Frequent Detection of HIV-1 Variants With Mixed Coreceptor Usage Among People Who Inject Drugs Infected With CRF01_AE: Possible Association With Coreceptor Switch

**DOI:** 10.1093/ofid/ofag080

**Published:** 2026-02-21

**Authors:** Yosuke Maeda, Takayuki Chikata, Takeo Kuwata, Hiromi Terasawa, Giang Van Tran, Shuzo Matsushita, Tomohiro Sawa, Futoshi Hasebe, Masafumi Takiguchi

**Affiliations:** Department of Microbiology, Faculty of Life Sciences, Kumamoto University, Kumamoto, Japan; Department of Nursing, Kibi International University, Takahashi, Japan; Joint Research Center for Human Retrovirus Infection, Kumamoto University, Kumamoto, Japan; AIDS Clinical Center, National Center for Global Health and Medicine, Japan Institute for Health Security, Tokyo, Japan; Joint Research Center for Human Retrovirus Infection, Kumamoto University, Kumamoto, Japan; Department of Microbiology, Faculty of Life Sciences, Kumamoto University, Kumamoto, Japan; Joint Research Center for Human Retrovirus Infection, Kumamoto University, Kumamoto, Japan; National Hospital of Tropical Diseases, Hanoi, Vietnam; Joint Research Center for Human Retrovirus Infection, Kumamoto University, Kumamoto, Japan; Department of Microbiology, Faculty of Life Sciences, Kumamoto University, Kumamoto, Japan; Institute of Tropical Medicine, Nagasaki University, Nagasaki, Japan; Joint Research Center for Human Retrovirus Infection, Kumamoto University, Kumamoto, Japan

**Keywords:** coreceptor, CRF01_AE, HIV-1, mixed infection, people who inject drugs

## Abstract

**Background:**

Our previous study suggested that mixed infection with R5 and X4/dual human immunodeficiency virus type 1 (HIV-1) may contribute to coreceptor switch from R5 to X4 HIV-1. To confirm this hypothesis, we investigated mixed HIV-1 infections in people who inject drugs (PWID) infected with the CRF01_AE subtype.

**Methods:**

Viral plasma RNA from PWID were extracted, the V3 region of the HIV-1 gp120 gene was amplified, and deep sequencing was performed. Coreceptor usage was determined using phenotypic assay by cloning each V3 region. Coreceptor usage of minor HIV-1 variants detected by deep sequencing was predicted based on the amino acid sequences of the V3 region.

**Results:**

Deep sequencing of plasma from 36 PWID revealed that mixed HIV-1 infection involving different coreceptor usage occurred in 13 cases (36.1%). Phylogenetic analysis revealed that R5 variants were dominant, whereas X4/dual variants were detected as minor populations in most cases. In 1 case, however, R5 variants emerged as a distinct minor population mixed with X4/dual variants as the major population. Notably, plasma viral RNA load (pVL) was higher in cases of mixed infection with R5 and X4/dual HIV-1 than in those infected solely with R5 HIV-1.

**Conclusions:**

Our observations suggest a possible association between mixed HIV-1 coreceptor usage and coreceptor switch in CRF01_AE–infected PWID, and that mixed infection may be associated with pVL.

## Teaser

This study investigated coreceptor switch from R5 to X4 HIV-1 in CRF01_AE-infected people who inject drugs. We found a high frequency of mixed infection with R5 and X4 HIV-1 and an association between mixed infection with elevated plasma viral load.

Approximately 38 million people worldwide have been diagnosed with human immunodeficiency virus (HIV). HIV exhibits high genetic diversity, with HIV type 1 (HIV-1) group M being the most prevalent. This group can be further divided into 9 distinct subtypes (A, B, C, D, F, G, H, J, and K) and various circulating recombinant forms (CRFs). To date, 178 CRFs have been documented in the Los Alamos database (https://www.hiv.lanl.gov/components/sequence/HIV/crfdb/crfs.comp) [1]. The CRF01_AE subtype is dominant in Southeast Asia and China [2] and is associated with accelerated disease progression compared to other subtypes and CRFs [3–8]. Generally, the cellular tropism and pathological phenotype of HIV-1 is largely determined by the usage of 2 coreceptors, CCR5 and CXCR4. HIV-1 variants that use only CCR5, referred to as R5 HIV-1, primarily infect CD4 T cells and macrophages and persist as the dominant HIV-1 form throughout the course of the infection. In contrast, HIV-1 variants that use only CXCR4, referred to as X4 HIV-1, primarily infect CD4 T cells and T-cell lines, and emerge in the later phase of infection with accelerated disease progression [9–11]. HIV-1 variants that use both CCR5 and CXCR4, referred to as R5X4 or dual HIV-1, also contribute to disease progression.

Accelerated disease progression is correlated with the emergence of CXCR4-tropic HIV-1 (referred to as X4/dual), a phenomenon that has been extensively studied in subtype B HIV-1 [9–11]. The CRF01_AE subtype has been reported to have a high prevalence of X4/dual HIV-1 [12, 13], which may account for the accelerated disease progression. However, the correlation between the high prevalence of X4/dual HIV-1 and faster disease progression in CRF01_AE infection remains poorly understood. Replication-competent X4 variants from the plasma of individuals infected with CRF01_AE, including those who dominantly carried R5 variants, have been isolated previously [14]. This previous study used deep sequencing analysis and identified X4 variants as the minor population and R5 variants as the major population in the plasma [14], implying that X4/dual variants had the potential to become dominant in later stages of infection. Therefore, longitudinal studies to monitor these cases over time are essential. However, analyzing the natural course of HIV-1 infection is challenging due to the widespread use of antiretroviral therapy. Additional deep sequencing analyses of HIV-1 mixed with R5 and X4/dual variants are needed to elucidate the role of coreceptor switch. Previous research identified only 3 cases of mixed variants with different coreceptor usage. People who inject drugs (PWID) are exposed to multiple variants of HIV-1 at different times, and have a higher frequency of X4/dual HIV-1 infection in their plasma, likely due to the direct transmission via blood [15]. This study included PWID to increase the number of cases with the mixed tropism to investigate the role of mixed infections in coreceptor switch.

## MATERIALS AND METHODS

### Study Participants and Ethics Statement

A total of 54 treatment-naive Vietnamese PWID chronically infected with CRF01_AE HIV-1 were recruited from the National Hospital of Tropical Diseases, Vietnam, from 2012 to 2015. Drug use was determined based on the participant's declaration. All participants were HIV-1 antibody positive on immunoassay when the samples were prepared. Written informed consent was provided by all participants in accordance with the Declaration of Helsinki. The study protocol was approved by the Institutional Review Board of the National Hospital of Tropical Diseases, the Vietnamese Ministry of Health (1666/QĐ-BYT), and the Ethic Committees for Epidemiology and General Study in the Faculty of Life Sciences at Kumamoto University (RINRI-1340 and GENOME-342).

### Cell Lines and Culture Conditions

The human embryonic kidney cell line HEK-293T was cultured in Dulbecco's modified Eagle medium (Sigma-Aldrich, St Louis, Missouri, USA) supplemented with 10% heat-inactivated fetal bovine serum (FBS; Thermo Fisher Scientific, Waltham, Massachusetts, USA) and penicillin-streptomycin (PS; Nacalai Tesque, Kyoto, Japan). The CD4-expressing glioma cell line NP-2/CD4 was cultured in minimum essential medium (MEM; Sigma-Aldrich) supplemented with 10% FBS, PS, and G418 (0.1 mg/mL; Nacalai Tesque). NP-2/CD4 cells expressing either CCR5 or CXCR4 were cultured in MEM supplemented with 10% FBS, PS, G418 (0.1 mg/mL), and zeocin (10 μg/mL; Thermo Fisher Scientific), as previously described [16].

### Cloning of the V3 Region of HIV-1 gp120 and Prediction of Coreceptor Usage

Viral RNA was extracted from the plasma of CRF01_AE-infected PWID using the QIAmp Blood Mini kit (Qiagen, Hilden, Germany). Complementary DNA (cDNA) was synthesized from viral RNA (vRNA) using SuperScript IV Reverse Transcriptase (Thermo Fisher Scientific) with random hexamers, according to the manufacturer's instruction. The V3 spanning region of HIV-1 gp120 was amplified by nested polymerase chain reaction (PCR) using high-fidelity DNA polymerase, PrimeSTAR HS, as previously described [14]. The first PCR used forward primer 5′- TCACARACAATGCC-3′ (VI-F5N) and reverse primer 5′-CCCTCTACAATTAAAATGATG-3′ (VI-R4). The second PCR used forward primer 5′-CCATAATAGTGCACCT-3′ (VI-F6N) and reverse primer 5′-TAGATCTCCTCCTGAGG-3′ (VI-R2). The amplified products were then cloned into a TOPO vector (Thermo Fisher Scientific) for sequencing using the Applied Biosystems 3130 Genetic Analyzer (Thermo Fisher Scientific). Coreceptor usage was predicted based on the amino acid sequences of the V3 region. The combined rule by Raymond et al [17], including the “11/25 rule,” where HIV-1 was predicted as CXCR4-using HIV-1 if charged amino acids were observed at positions 11 or 25 of the V3 region [17]; net charge (NC) of the V3 region, calculated by subtracting the number of negatively charged amino acids (D and E) from positively charged ones (K and R); and the presence of potential N-glycan site (PNGS) between amino acids 6 and 8 in the V3 region determined by the presence of amino acid sequence NXT/S (with X representing any amino acid). In brief, 1 of the following criteria predicted the CXCR4 usage: (*i*) 11R/K and/or 25K in V3; (*ii*) 25R in V3 and NC of ≥+5; (*iii*) NC of ≥+6; (*iv*) loss of PGNS and NC of ≥+4. The Geno2Pheno_[coreceptor]_ algorithm (https://coreceptor.geno2pheno.org/) was used with a false-positive rate (FPR) [18].

### Phenotypic Assay for Coreceptor Usage

Phenotypic analysis of coreceptor usage was performed using pseudotyped HIV-1 carrying the V3 region cloned from the plasma vRNA as previously described [14]. In brief, the amplified V3 region cloned from the plasma vRNA was introduced into the *AflII* and *NheI* cloning sites of an Env expression vector, pCXN-FLan [19]. A luciferase reporter HIV-1 pseudotyped with Env carrying the cloned V3 region was produced by co-transfection of HEK-293T cells with pNL-LucΔ*BglII* and each Env expression vector [19] using Lipofectamine 2000 (Thermo Fisher Scientific). NP-2/CD4 cells expressing either CCR5 or CXCR4 were then infected with equal amounts of the pseudotyped HIV-1 (10 ng p24 Ag) and cultured for 48 hours. Luciferase activity within the infected cells was measured using the One-Glo Luciferase Assay System (Promega, Fitchburg, Wisconsin, USA) and GloMax Luminometer (Promega).

### Deep Sequencing of the V3 Region in Plasma vRNA

The cDNA synthesized from plasma vRNA was first amplified using the primers VI-F5N and VI-R4, as described above. A second PCR was performed using forward primer 5′-ACACTCTTTCCCTACACGACGCTCTTCCGATCTCCATAATAGTGCACCT-3′ and reverse primer 5′-GTGACTGGAGTTCAGACGTGTGCTCTTCCGATCTTAGATCTCCTCCTGAGG-3′. Adapter sequences for deep sequencing library preparation are underlined. Library preparation and deep sequencing were performed by FASMAC (https://fasmac.co.jp/en) (Kanagawa, Japan) using MiSeq (Illumina, San Diego, California, USA).

### Phylogenetic Analysis

Deep sequencing V3 region data exceeding 0.1% frequency were aligned using CLC Sequence Viewer version 7.7.1 (Qiagen, Redwood City, California, USA). A phylogenetic tree was constructed using the neighbor-joining method with the Jukes–Cantor distance model (bootstrap 1000 replicates) using CLC Sequence Viewer version 7.7.1 (Qiagen).

### Statistical Analysis

One-way analysis of variance (ANOVA) and nonparametric 2-tailed Mann–Whitney *U* test were performed using GraphPad Prism version 10.1.0 (GraphPad Software, San Diego, California, USA).

## RESULTS

### Validation of Prediction Methods for CRF01_AE HIV-1 Coreceptor Usage

In a previous study, replication-competent X4 variants were isolated from the plasma of CRF01_AE-infected individuals in 3 of 30 cases, even in those who displayed the R5 variants as the major population [14], suggesting that HIV-1 variants mixed with different coreceptor usage may play a role in the coreceptor switch from CCR5 to CXCR4. To this end, the precise prediction of coreceptor usage of minor HIV-1 variants without phenotypic assays should be established. The Geno2Pheno_[coreceptor]_ algorithm [18] has been commonly used to predict coreceptor usage in subtype B HIV-1, the predominant strain in Western Europe and the United States. However, this algorithm has proved less efficient for coreceptor prediction in other subtypes, such as subtype C and CRF01_AE [17, 20–22]. Raymond et al reported that the most accurate method for predicting CRF01_AE coreceptor usage involves combining the 11/25 rule, NC rule, and PNGS mutation analysis [17]. Therefore, the present study validated the Geno2Pheno_[coreceptor]_ algorithm and the combined rule by Raymond et al using phenotypic assay. To enhance the accuracy of the method, the data of phenotypic assay of the present study were analyzed in combination with previous datasets [14]. In brief, major V3 region sequences from the Illumina libraries were molecularly cloned into an Env expression vector, and coreceptor usage of each clone was determined using NP-2/CD4, expressing either CCR5 or CXCR4, as previously described [14]. For the Geno2Pheno_[coreceptor]_ algorithm, an FPR of <2.5% was selected to predict CXCR4 usage, such as X4 or dual HIV-1 (Supplementary Table 1). Both tools exhibited 100% sensitivity, but the combined rule demonstrated higher specificity (98%) than the Geno2Pheno_[coreceptor]_ algorithm (92%) in the present study. The concordance rates of Geno2Pheno_[coreceptor]_ and the combined rule were 94% and 99%, respectively. Therefore, the combined rule for genotypic prediction of coreceptor usage was selected for the present study.

### Deep Sequencing of the V3 Region to Identify HIV-1 Mixed With R5 and X4/Dual Variants in CRF01_AE-Infected PWID

To clarify the role of mixed HIV-1 with different coreceptor usage in the coreceptor switch, we focused on PWID infected with the CRF01_AE subtype. Generally, HIV-1 transmission is mediated by R5 HIV-1 in humans, with X4/dual HIV-1 emerging later through the evolution from R5 HIV-1 at a later stage of infection. However, PWID are frequently exposed to multiple HIV-1 variants from various individuals at different times, leading to more frequent and direct transmission of X4/dual HIV-1 via the blood [15]. Therefore, we hypothesized that PWID plasma would be suitable for detecting HIV-1 mixed with R5 and X4/dual variants.

To identify minor populations of HIV-1 variants, Illumina libraries carrying the V3 region were generated from plasma vRNA of CRF01_AE-infected PWID using nested PCR. All 54 samples were subjected to amplify the V3 region for the preparation of Illumina libraries, while only 36 cases were successfully amplified. The number of total reads of Illumina libraries exceeded 1 × 10^6^ reads in the plasma of all infected individuals, and those containing >0.1% of the total reads were further analyzed to exclude the PCR-mediated error. Amino acid sequence of the V3 region of each subject was determined, and coreceptor usages of major sequences, which were molecularly cloned, were confirmed by our phenotypic assay. Coreceptor usages of minor sequences of the V3 region, which were not molecularly cloned, were predicted using the combined rule as previously described (Supplementary Table 1).

**Figure 1.**
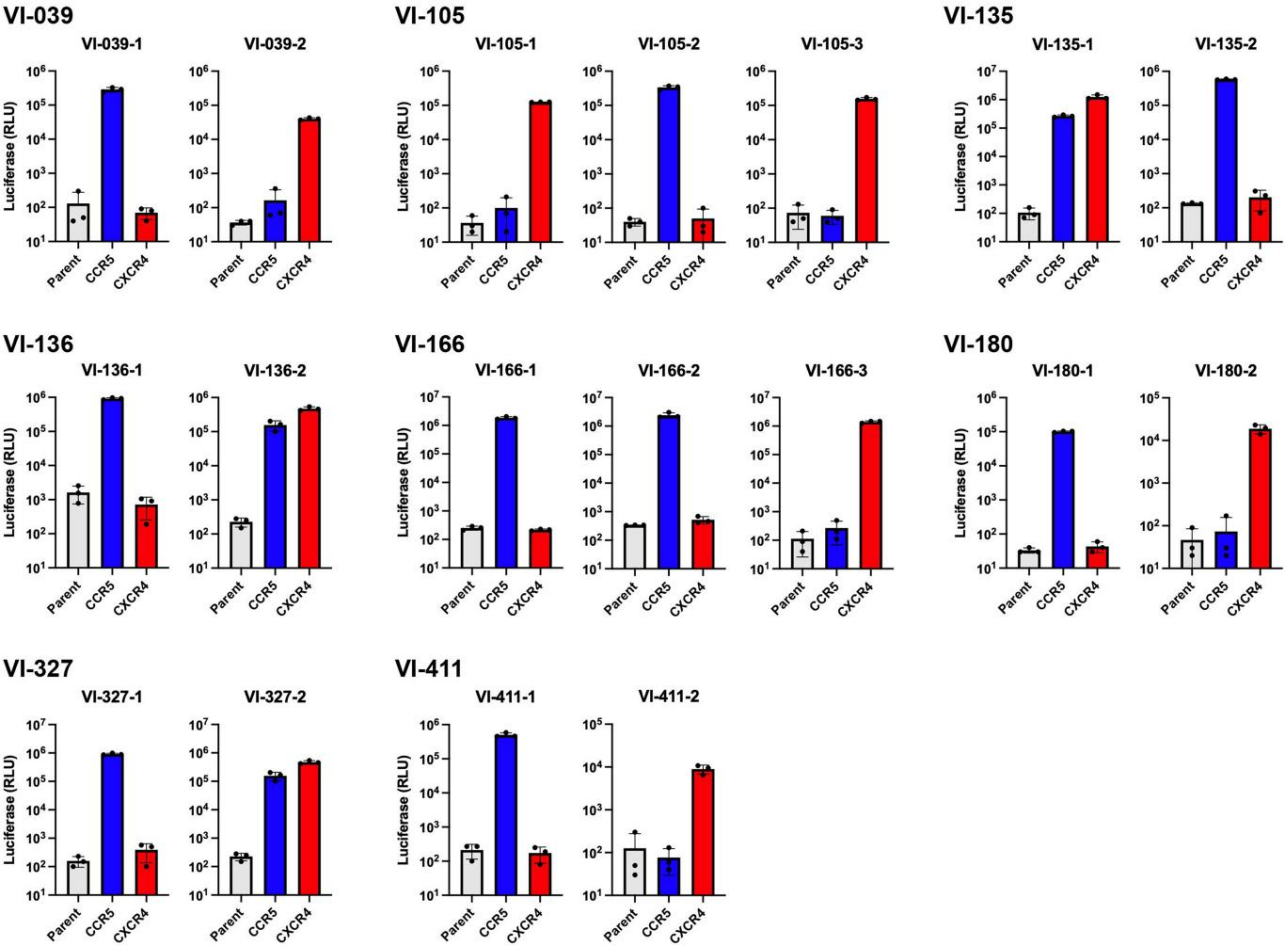
Coreceptor usage of V3 region of HIV-1 cloned from plasma viral RNA (vRNA) by phenotypic assay. NP-2/CD4 cells (parent) and NP-2/CD4 cells expressing CCR5 and CXCR4 were infected with the same amount of luciferase reporter pseudotyped virus carrying each V3 cloned from plasma vRNA of people who inject drugs infected with CRF01_AE (10 ng p24 Ag). Luciferase activities were measured at 48 hours postinfection. Luciferase activities of 2–3 V3 regions carrying different amino acid sequences from the same individuals are shown according to Table 1. Data are geometric mean ± standard deviation of triplicate experiments.

Using deep sequencing, 13 of 36 cases (36.1%) with the mixed infection showing different coreceptor usage were identified, 8 of which were confirmed using the phenotypic assay (Figure 1 and Table 1). The other 5 cases were identified using genotypic assay alone (Supplementary Table 2). Among the cases confirmed by phenotypic assay, 5 cases exhibited a mixture of R5 and X4 variants, whereas 3 cases showed a mixture of R5 and dual variants (Figure 1 and Table 1). In addition, 19 cases (52.8%) harbored R5 variants alone, whereas 4 cases (11.1%) harbored X4 variants alone (Supplementary Table 3). In total, X4/dual variants were identified in 17 of 36 cases (47.2%), indicating a high frequency of X4/dual variants when deep sequencing was applied (summarized in Table 2).

**Table 1.** Virological Analysis of the V3 Region of HIV-1 Mixed With R5 and X4/Dual Confirmed by Phenotypic Assay

ID	V3 Region Sequence^a^	Frequency, %	Length	11/25 aa^b^	PNGS	NC	FPR, %	Genotype^c^	Phenotype^d^
VI-039	CTRPSNNTRTGIHIGPGR-VFYRTGEITGDIRKAYC	73.1	35	G/E	+	+5	6.9	R5	R5
	……….S….L..R.Y….R.I……..	26.9	36	S/R	+	+7	1.5	X4/dual	X4
VI-105	CTRPTTSKRKRITMGPGRVYYSTKQIVGDIKKAQC	69.0	35	R/Q	−	+8	0.1	X4/dual^e^	X4
	….SNNT..SM.I…Q.F.R.GD.I…R..Y.	22.4	35	S/D	+	+3	13.0	R5	R5
	….SYTEI.-M.R…H.F.R.GK.I…R..Y.	7.2	34	K/K	−	+5	0.5	X4/dual	X4
	….SNNT..GM.I…Q.F.R.GD.I…R..Y.	1.4	35	G/D	+	+4	10.5	R5	ND
VI-135	CTRPSNNTRKRMTLGPGRVFYSTGEIIGDIRKAFC	49.7	35	R/E	+	+5	0.2	X4/dual	X4
	………TS.PI…Q…R..D…N….Y.	49.7	35	S/D	+	+4	60.9	R5	R5
	……………..Q…R..D…N….Y.	0.2	35	R/S	+	+6	1.1	X4/dual	ND
	……….G……………………	0.1	35	G/E	+	+4	2.6	R5	ND
VI-136	CTRPGNNTRKSTNIGPGQVLFYRPGDIIGDIKKAHC	79.6	36	S/D	+	+4	27.1	R5	R5
	….-YK.KTGVTR.L.R.-…T.EVE…R.TY.	20.4	34	G/E	−	+5	0.5	X4/dual	Dual
VI-166	CTRPSNNTRTSIPMGPGRAFYRTGDIIGDIRKAYC	43.6	35	S/D	+	+4	41.3	R5	R5
	……..I.G.TI….V…….R……..	27.7	35	G/D	+	+4	8.5	R5	R5
	….Y.Y..VRVT…..VY….EIR…K….	28.7	35	R/E	+	+6	0.2	X4/dual	X4
VI-180	CTRLDNNTRKSISVGPGRIYYRPGDIIGDIRKAHC	37.1	35	S/D	+	+3	9.0	R5	R5
	…PNLS.K..MI..T…L..T.KV….K..Y.	29.0	35	S/K	+	+6	1.7	X4/dual	X4
	…PS….T.LT.A..QVL..T……….Y.	33.9	35	S/D	+	+4	6.9	R5	ND
VI-327	CTRPSNNTRKRMTLGPGRVFYSTGEIIGDIRKAYC	97.3	35	R/E	+	+5	0.4	X4/dual	Dual
	……..IIS..I…Q…R..D……….	2.5	35	S/D	+	+2	11.4	R5	R5
	……….G……………………	0.1	35	G/E	+	+4	2.9	R5	ND
VI-411	CTRPDNNTRKSVNVGPGQVLFYKSGDIIGNIRKAYC	37.7	36	S/D	+	+6	8.5	R5	R5
	….G….R.I.I……..RP..V……..C	39.1	36	S/D	+	+5	38.0	R5	ND
	….SGQRKTRITM…R.-..RT.E.V.S.K….	23.3	35	R/E	−	+6	0.7	X4/dual	X4

Abbreviations: FPR, false-positive rate in Geno2Pheno_[coreceptor]_; HIV-1, human immunodeficiency virus type 1; NC, net charge; ND, not determined; PNGS, potential N-linked glycosylation site.

^a^Dots denote sequence identity. Dashes denote absence of amino acid. Only representative amino acid sequences are shown.

^b^Amino acid residues at positions 11 and 25 of the V3 region.

^c^Genotype: coreceptor usage of the V3 region, as determined using the combined rule with 11/25, NC, and PNGS.

^d^Phenotype: coreceptor usage of each V3 region, as determined using pseudotype virus assay.

^e^X4/dual: CXCR4-using HIV-1 through genetic prediction.

**Table 2.** Viral Tropism With Clinical Data of People Who Inject Drugs Infected With HIV-1 CRF01_AE

ID	Age, y	Sex	pVL, Log_10_ Copies/mL	CD4 Count, Cells/μL	R5, %^a^	X4, %^a^	Tropism^b^ (Phenotype)
VI-031	33	M	4.06	184	0.0	100.0	X4
VI-039	34	M	3.93	238	73.1	26.9	Mixed
VI-054	49	M	4.78	655	100.0	0.0	R5
VI-063	32	M	5.18	93	96.0	4.0	Mixed
VI-066	38	M	4.12	975	100.0	0.0	R5
VI-067	43	M	4.92	4	100.0	0.0	R5
VI-074	35	M	4.84	88	100.0	0.0	R5
VI-081	28	M	4.78	463	100.0	0.0	R5
VI-086	34	M	4.94	412	100.0	0.0	R5
VI-096	28	M	4.98	324	100.0	0.0	R5
VI-105	27	M	4.05	150	43.2	56.8	Mixed
VI-106	62	M	5.52	50	94.3	5.7	Mixed
VI-108	40	M	5.14	406	100.0	0.0	R5
VI-109	30	M	4.93	177	0.0	100.0	X4
VI-116	30	M	5.12	8	83.7	16.3	Mixed
VI-120	33	M	4.76	328	100.0	0.0	R5
VI-135	29	M	4.97	262	50.1	49.9	Mixed
VI-136	28	M	5.53	333	79.6	20.4	Mixed
VI-166	27	M	5.73	9	71.3	28.7	Mixed
VI-180	32	M	5.83	272	71.0	29.0	Mixed
VI-185	27	F	4.76	390	0.0	100.0	X4
VI-213	16	M	4.51	364	100.0	0.0	R5
VI-219	28	M	4.72	545	100.0	0.0	R5
VI-290	26	M	5.27	181	97.6	2.4	Mixed
VI-301	30	M	4.07	75	0.0	100.0	X4
VI-309	35	M	5.09	8	100.0	0.0	R5
VI-310	28	M	3.95	310	100.0	0.0	R5
VI-313	36	M	4.93	9	100.0	0.0	R5
VI-327	24	M	4.41	467	2.7	97.3	Mixed
VI-333	48	M	5.56	87	89.0	11.0	Mixed
VI-404	26	M	4.64	230	100.0	0.0	R5
VI-410	29	M	5.10	439	100.0	0.0	R5
VI-411	30	M	4.94	100	76.7	23.3	Mixed
VI-421	35	M	5.10	111	100.0	0.0	R5
VI-527	32	M	4.94	248	100.0	0.0	R5
VI-535	37	M	4.86	186	100.0	0.0	R5

Abbreviations: F, female; HIV-1, human immunodeficiency virus type 1; M, male; pVL, plasma viral load.

^a^The frequency of R5 and X4/dual HIV-1 in each plasma of injection drug users was determined by the numbers of reads obtained from Illumina libraries of the V3 region.

^b^Tropism of the V3 region of the major populations of the reads was determined by the phenotypic assay, whereas the tropism of minor population of the reads, which was not confirmed by the phenotypic assay, was predicted by the combined rule with 11/25, net charge, and potential N-linked glycosylation site mutation.

**Figure 2.**
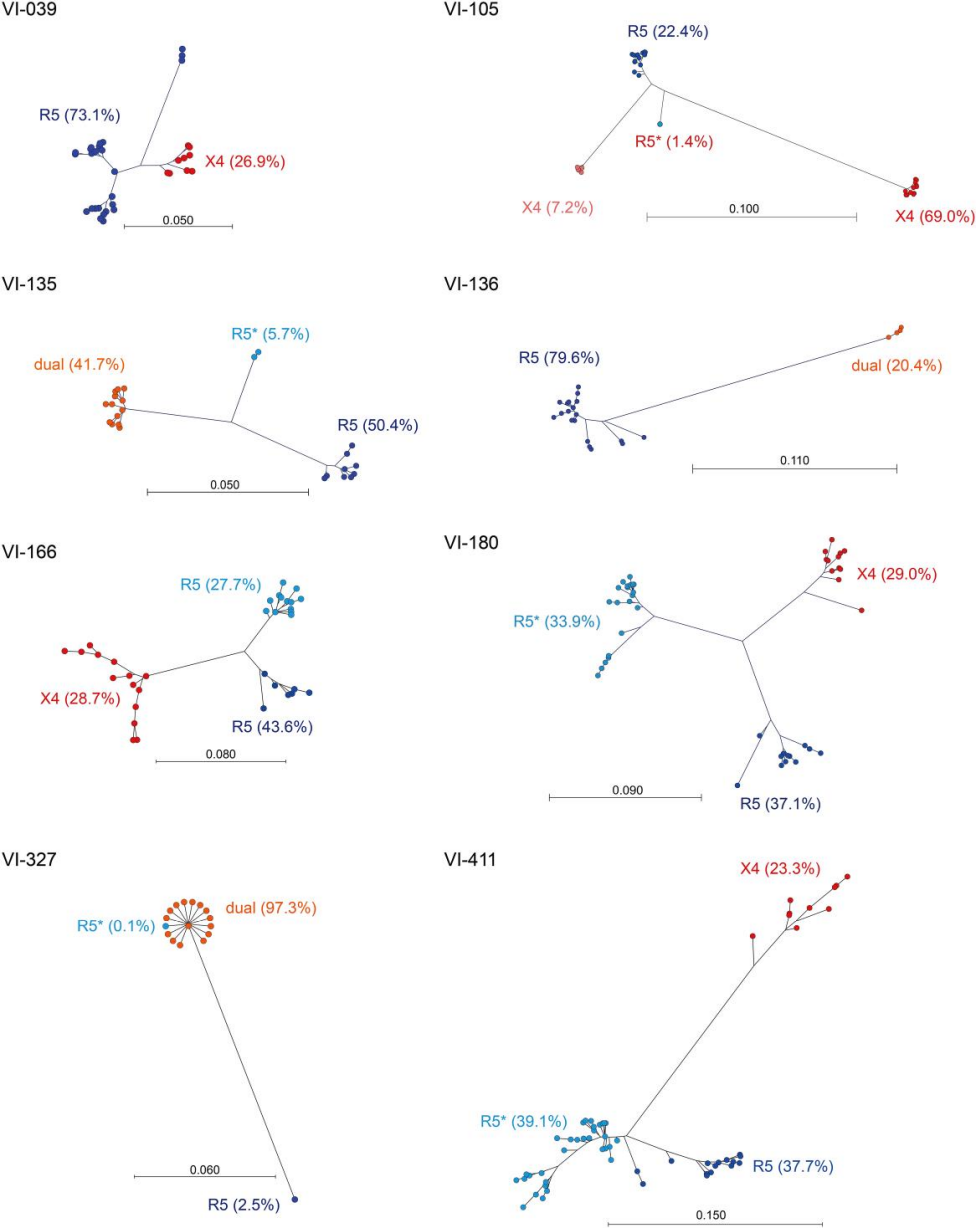
Phylogenetic trees of HIV-1 mixed with R5 and X4/dual variants confirmed by phenotypic assay. Phylogenetic trees were constructed based on sequences representing >0.1% of reads from Illumina libraries carrying the V3 region, using the neighbor-joining method and Jukes–Cantor distance model. Coreceptor usage of clusters identified using genotypic assay alone is marked with an asterisk (*). Blue and light blue dots represent the clusters of R5 variants. Red and light red dots represent the clusters of X4/dual variants. The scale bar represents genetic distance.

To identify the origin of R5 and X4/dual variants in the mixed tropism cases, phylogenetic trees of the 13 mixed tropism cases were constructed (8 cases confirmed by phenotypic assay) (Figure 2), whereas 5 cases predicted by genetic analysis alone (Supplementary Figure 1). Both analyses revealed that R5 and X4/dual HIV-1 clusters were genetically distant. Furthermore, most of mixed tropism cases displayed that R5 variants populations were dominant, whereas X4/dual variants populations were less abundant or minor except cases VI-105 and VI-327 (Figure 2). Especially, in case VI-327, R5 HIV-1 clusters were observed as the minor population, which were genetically distant from the major population of dual HIV-1 clusters (Figure 2). In this case, 1 cluster within the major dual HIV-1 population was predicted to be R5 variants based on genotypic analysis (Figure 2). These results indicated the high frequency of mixed infection with R5 and X4/dual in CRF01_AE-infected PWID.

### Elevated Plasma Viral Load in the Mixed Tropism Cases With R5 and X4/Dual Variants

**Figure 3.**
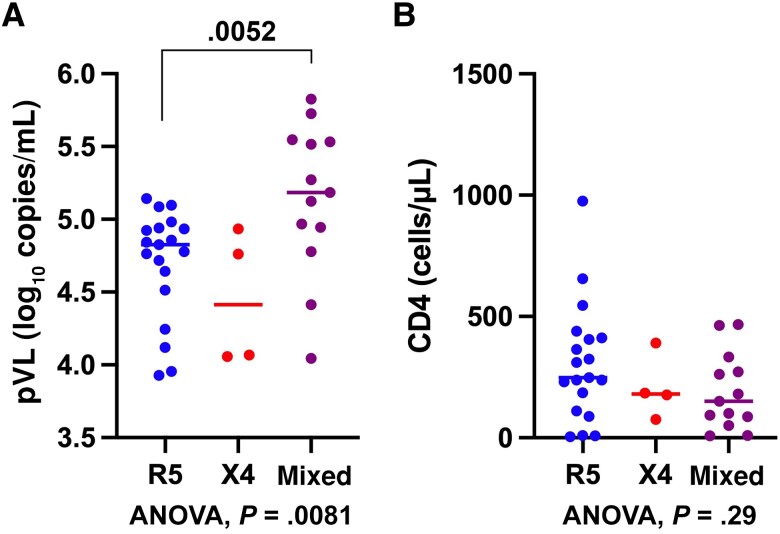
Plasma viral load (pVL) and CD4 T-cell count in individuals harboring R5 alone, X4 alone, and mixed with R5 and X4/dual variants. The pVL (log copies/mL) (*A*) and CD4 T-cell count (cells/μL) (*B*) are shown with the median and interquartile range for each group. Significant differences in pVL and CD4 count are determined using 1-way analysis of variance (ANOVA) with *P* values shown at the bottom of each panel. The *P* value of nonparametric 2-tailed Mann–Whitney *U* test is shown between R5 and mixed tropism in (*A*).

This study further examined whether the mixed tropism cases harboring R5 and X4/dual variants were correlated with clinical parameters, such as plasma viral load (pVL) and CD4 T-cell count shown in Table 2, compared to single tropism cases harboring R5 and X4 variants alone (summarized in Supplementary Table 4). One-way ANOVA showed a significant difference (*P* = .0081) in pVL among these individuals, whereas pVL in individuals infected with R5 variants alone was significantly lower than those infected with a mixture of R5 and X4/dual variants (Figure 3*A*). In contrast, no significant difference was shown in CD4 T-cell count between these groups (Figure 3*B*).

## DISCUSSION

The mechanism underlying the switch of coreceptor usage from CCR5 to CXCR4 during the later stage of natural HIV-1 infection remains unclear. Generally, the susceptibility of CD4 T-cell subsets to R5 or X4/dual HIV-1 is thought to depend on the expression levels of CCR5 and CXCR4 within each T-cell subset. For example, CXCR4 is consistently expressed across most CD4 T-cell subsets, while CCR5 expression relies on the T-cell subsets, with higher CCR5 expression in memory T cells than in naive T cells [23–25]. In the early stages of infection, R5 HIV-1 predominantly infects and destroys memory CD4 T cells. Although CD4 T-cell homeostasis is maintained through rapid cell turnover, memory CD4 T-cell depletion can occur with sustained infection. Consequently, a shift in cell tropism occurs to allow HIV-1 variants to use the CXCR4 receptor by substituting an amino acid sequence of the V3 region. These findings have been obtained in studies using subtype B HIV-1, which is most prevalent in Western Europe and the United States; however, studies investigating coreceptor switch in CRF01_AE subtype, which predominantly circulates in Southeast Asia [2] with high frequency of X4/dual HIV-1 variants, are limited [12].

In a previous study, 3 cases carrying HIV-1 mixed with R5 and X4 variants were identified among 30 cases in the CRF01_AE subtype using deep sequencing [14]. Notably, X4 variants were isolated from the plasma of 2 cases where conventional Sanger sequencing of the V3 region only identified the R5 variants, while 1 case showed the mixed tropism with R5 and X4 variants using Sanger sequencing. These findings suggest that the coreceptor switch from CCR5 to CXCR4 may partly occur via long-term coexistence of R5 and X4/dual variants without stepwise transition through intermediates [26]. Indeed, selective pressures, such as CCR5 inhibitors, can readily select X4/dual variants in cases of HIV-1 mixed with R5 and X4/dual variants in vivo [27, 28]. This suggests that the long-term coexistence of R5 and X4/dual HIV-1 variants in the same individual may induce coreceptor switch from CCR5 to CXCR4 in response to some selective pressures, such as humoral immune responses [29, 30] or environmental changes in replication fitness of different HIV-1 variants.

To further elucidate the role of HIV-1 mixed with R5 and X4/dual variants in coreceptor switch in the CRF01_AE subtype, we analyzed cases carrying HIV-1 variants mixed with different coreceptor usage in the present study using plasma samples from PWID infected with CRF01_AE subtype, as PWID are likely exposed to multiple HIV-1 variants over time. Indeed, 1 case harboring major R5 and minor X4 variants in a previous study involved a PWID [14]. Recent deep sequencing approaches provide improved sensitivity to detect minor populations such as X4/dual variants in major R5 variant populations, as previously described [13, 31–43]. In the present study, 13 cases harboring X4/dual mixed with R5 variants from 36 CRF01_AE-infected individuals were identified using deep sequencing (36.1%). However, whether the high frequency of mixed tropism was attributable to PWID or the CRF01_AE subtype remains unclear. Most of the X4/dual variants were identified as the less abundant or minor populations (11 of the 13 mixed tropism cases), whereas 2 cases (VI-105 and VI-327) displayed X4/dual variants as the major populations. Among the 2 cases carrying X4/dual variants as the major population, phylogenetic analysis of VI-327 showed minor R5 HIV-1 clusters genetically distant from the major dual HIV-1 clusters (Figure 1), which may represent the remnants of the previous major population prior to the coreceptor switch from CCR5 to CXCR4. Taken together, these findings suggest that, in some cases, a coreceptor switch of the CRF01_AE subtype from CCR5 to CXCR4 may occur in the context of long-term coexistence of HIV-1 variants with different coreceptor usage. However, because the duration of infection in these individuals is unknown and longitudinal data are not available, these observations should not be interpreted as evidence of a definitive switching mechanism. Finally, the hypothesis of the present study could not be fully validated due to the small number of cases, the unknown duration of infection, and the lack of longitudinal data. Further cross-sectional and longitudinal studies will be required to clarify the mechanisms underlying coreceptor switching in CRF01_AE infection.

Alongside the high frequency of mixed HIV-1 variants with different coreceptor usage in CRF01_AE-infected PWID, individuals harboring mixed with R5 and X4/dual variants were found to have a higher pVL compared with those infected with R5 variants alone. Although a larger size of analysis is required to verify the validity of this data, a previous in vitro study has demonstrated that X4 HIV-1 augmented R5 HIV-1 infection [44] by increasing the number of R5 HIV-1-infected cells, implying a potential synergistic effect between R5 and X4/dual HIV-1. Alternatively, R5 and X4/dual HIV-1 may infect different CD4 T-cell subsets, resulting in an overall increase in the number of infected cells.

In the present study, CD4 T-cell counts did not differ significantly between individuals harboring R5 and X4/dual variants and those infected with R5 variants alone. Since the present study identified only 4 cases harboring X4 variants alone, it was impossible to clarify whether CXCR4 tropims or mixed tropisms in the CRF01_AE subtype contribute to disease progression. Previous studies have linked CRF01_AE HIV-1 infection with accelerated disease progression, potentially due to the high prevalence of X4/dual HIV-1 [3–5, 7]. In addition, recent studies have also shown that several phylogenetic clusters of CRF01_AE preferentially using CXCR4 were correlated with CD4 T-cell loss [45, 46], although these studies did not examine mixed tropism with different coreceptor usage. The findings of the present study imply that the coexistence of R5 and X4/dual in HIV-1–infected individuals may play a role in the pathogenesis of the CRF01_AE subtype by activation or expansion of X4/dual variant–infected cells through long-term infection with R5 variants. Future studies are needed to elucidate this hypothesis.

Overall, it remains yet to be determined whether the high frequency of mixed tropism with different coreceptor usage is specific to PWID or the CRF01_AE subtype. Therefore, future studies of the mixed tropism cases in non-PWID such as sexual or maternal transmission–infected individuals with CRF01_AE or other subtypes may further elucidate the roles of coexistence of CCR5- and CXCR4-tropic HIV-1 in coreceptor switch and HIV-1 pathogenesis.

## Supplementary Material

ofag080_Supplementary_Data

## Supplementary Data

Supplementary materials are available at *Open Forum Infectious Diseases* online. Consisting of data provided by the authors to benefit the reader, the posted materials are not copyedited and are the sole responsibility of the authors, so questions or comments should be addressed to the corresponding author.
