# Supplementary material for: Frequent Detection of HIV-1 Variants With Mixed Coreceptor Usage Among People Who Inject Drugs Infected With CRF01_AE: Possible Association With Coreceptor Switch
Source: Open Forum Infect Dis. 2026 Feb 21;13(2):ofag080. doi: 10.1093/ofid/ofag080 (PMC12951246; doi:10.1093/ofid/ofag080)
Supplement: ofag080_Supplementary_Data [file ofag080_supplementary_data.zip › OFID_supplemental_figure_legends.docx]

**Supplmental figure legends**

**Figure S1: Phylogenetic trees of HIV-1 mixed with R5 and X4/dual variants predicted by genotypic assay alone.** Phylogenetic trees were constructed based on sequences representing >0.1 % of reads from Illumina libraries carrying the V3 region, using the neighbor joining method and Jukes–Cantor distance model. Coreceptor usage of clusters identified using genotypic assay alone is marked with an asterisk (*). Blue and light blue dots represent the clusters of R5 variants. Red and light red dots represent the clusters of X4/dual variants predicted by the combined rule. The scale bar represents genetic distance.
