# Supplementary material for: Frequent Detection of HIV-1 Variants With Mixed Coreceptor Usage Among People Who Inject Drugs Infected With CRF01_AE: Possible Association With Coreceptor Switch
Source: Open Forum Infect Dis. 2026 Feb 21;13(2):ofag080. doi: 10.1093/ofid/ofag080 (PMC12951246; doi:10.1093/ofid/ofag080)
Supplement: ofag080_Supplementary_Data [file ofag080_supplementary_data.zip › Table_S1.docx]

Table S1. Genotypic prediction of coreceptor usage of CRF01_AE virus clones in northern Vietnam

|  |  | No. of clones with indicated phenotype | |  | Genotypic prediction | | | | |
| --- | --- | --- | --- | --- | --- | --- | --- | --- | --- |
| Genotypic tool | Virus | R5 | X4/dual |  | % Sen*^a^* | % Spe*^b^* | % PPV*^c^* | % NPV*^d^* | Concordance*^e^* (%) |
| Geno2pheno 2.5% | R5 | 60 | 0 |  | 100 | 92 | 78 | 100 | 94 |
|  | X4 | 5 | 18 |  |  |  |  |  |  |
|  |  |  |  |  |  |  |  |  |  |
| Combined 11/25, net charge, and PGNS*^f^* mutation | R5 | 64 | 0 |  | 100 | 98 | 95 | 100 | 99 |
|  | X4 | 1 | 18 |  |  |  |  |  |  |

*a* Sen, sensitivity for detection of X4/dual viruses, calculated as the number of concordant X4 results divided by the number of viruses confirmed as X4/dual; *^b^*Spe, specificity for detecting exclusive CCR5-using viruses, calculated by the number of concordant R5 results divided by the number of viruses confirmed as R5; *^c^*PPV, positive predictive value, calculated by the number of concordant X4/dual results (true positive) divided by the number of viruses predicted X4 by the genotypic algorithm (number of positive tests); *^d^*NPV, negative predictive value, calculated by the number of concordant R5 results (true negative) divided by the number of viruses predicted R5 with the genotypic algorithm (number of negative tests). *^e^*Concordance between each genotypic algorithm and the phenotype was calculated as follows: number of samples with a concordant R5 genotype and phenotype plus number of samples with a concordant X4/dual genotype and phenotype, all divided by the total number of tested samples. *^f^*PNGS, potential N-glycosylation site.
