# Supplementary material for: Frequent Detection of HIV-1 Variants With Mixed Coreceptor Usage Among People Who Inject Drugs Infected With CRF01_AE: Possible Association With Coreceptor Switch
Source: Open Forum Infect Dis. 2026 Feb 21;13(2):ofag080. doi: 10.1093/ofid/ofag080 (PMC12951246; doi:10.1093/ofid/ofag080)
Supplement: ofag080_Supplementary_Data [file ofag080_supplementary_data.zip › Table_S2.docx]

Table S2. Virological analysis of the V3 region of HIV-1 mixed with R5 and X4/dual phenotype predicted by genotypic assay alone

| ID | V3 region sequence*^a^* | Frequency (%) | Length | 11/25aa*^b^* | PNGS*^c^* | NC*^d^* | FPR*^e^*  (%) | Genotype*^f^* | Phenotype*^g^* |
| --- | --- | --- | --- | --- | --- | --- | --- | --- | --- |
| VI-063 | CFRPFNNTRKSTTIGPGQVFYRTGDIIGDIRKAYC  CI..S....T.ISM.....................  .I...HTI.R..P..Q...IHK.RQ.T........ | 86.2  9.9  4.0 | 35  35  35 | S/D  S/D  S/Q | +  +  - | +4  +3  +6 | 2.4  24.0  0.0 | R5  R5  X4/dual | R5  ND*^h^*  ND |
| VI-106 | CTRPSNNTRKGMTIGPGQVFYRTGDIIGDIRKAYC  ....T....TSI.....R......E........Q.  .....Y-.EIK..R...H......K.......... | 81.8  12.5  5.7 | 35  35  34 | G/D  S/E  K/K | +  -  - | +4  +4  +5 | 10.5  4.0  0.5 | R5  R5  X4/dual | R5  ND  ND |
| VI-116 | CTRPSNNTRKSISIGPGKVFYQTGDIIGNIRQAYC  .........TG.H....Q..........D..K...  ....A.K..TR.T....R.....E..R.D..K... | 64.9  18.8  16.3 | 35  35  35 | S/D  G/D  R/D | +  +  + | +4  +4  +5 | 15.0  42.3  0.1 | R5  R5  X4/dual | R5  R5  ND |
| VI-290 | CTRPSNNTRTSISMGPGRVFFGTGDIIGNIRKAHC  ....YK-.K.GVTR.L... YR..EVE.D...TY. | 97.6  2.4 | 35  34 | S/D  G/E | +  - | +4  +5 | 21.2  0.5 | R5  X4/dual | R5  ND |
| VI-333 | CTRPSNNTRRSVTIGPGQVFYRTGEIIGDIRQAYC  .........K.TN.........A.D...N......  ....FETI..RTA..Q...LSSAEA.K........ | 76.0  13.0  11.0 | 35  35  35 | S/E  S/D  R/A | +  +  - | +3  +4  +5 | 39.4  14.6  0.0 | R5  R5  X4/dual | R5  R5  ND |

*^a^*Dots denote sequence identity. Dashes denote absence of amino acid. Only representative amino acid sequences are shown; *^b^*Amino acid residues at positions 11 and 25 of the V3 region; *^c^*PNGS, potential N-linked glycosylation site; *^d^*NC, net charge; *^e^*FPR, false-positive rate in Geno2Pheno_[coreceptor]_; *^f^*Genotype, coreceptor usage of the V3 region, as determined using the combined rule with 11/25, net charge, and PNGS; *^g^*Phenotype, coreceptor usage of each V3 region, as determined using pseudotype virus assay; *^h^*X4/dual, CXCR4-using HIV-1 through genetic prediction; *^i^*ND, not determined.
