# Supplementary material for: Frequent Detection of HIV-1 Variants With Mixed Coreceptor Usage Among People Who Inject Drugs Infected With CRF01_AE: Possible Association With Coreceptor Switch
Source: Open Forum Infect Dis. 2026 Feb 21;13(2):ofag080. doi: 10.1093/ofid/ofag080 (PMC12951246; doi:10.1093/ofid/ofag080)
Supplement: ofag080_Supplementary_Data [file ofag080_supplementary_data.zip › Table_S3.docx]

Table S3. Virological analysis of the V3 region of HIV-1 with the same coreceptor usage

| ID | V3 region sequence*^a^* | Frequency (%) | Length | 11/25aa*^b^* | PNGS*^c^* | NC*^d^* | FPR*^e^*  (%) | Genotype*^f^* | Phenotype*^g^* |
| --- | --- | --- | --- | --- | --- | --- | --- | --- | --- |
| VI-054 | CTRPSNNTRKSIHMGPGRVFYRTGDIIGDIRKAYC  ............SV..................... | 68.2  31.8 | 35  35 | S/D  S/D | +  + | +5  +5 | 6.9  3.8 | R5  R5 | R5  R5 |
| VI-066 | CTRPANNTRTSINIGPGQVLFYRPGDVVGDIRKAYC  ....S...I...T......-...T..IK......F. | 66.1  33.9 | 36  35 | S/D  S/D | +  + | +3  +3 | 31.6  9.6 | R5  R5 | R5  R5 |
| VI-067 | CTRPSNNTRTGITMGPGRVFFRTGDVIRDIRKAYC  .....I....S.AI...K..Y....ITG....... | 99.4  0.6 | 35  35 | G/D  S/D | +  + | +5  +4 | 12.8  5.3 | R5  R5 | R5  ND*^h^* |
| VI-074 | CTRPANNTRTSINIGPGQVLFYRPGDVVGDIRKAYC  ........I...TM.....-...T.EIK........ | 52.6  47.4 | 36  35 | S/D  S/E | +  + | +3  +4 | 31.6  5.7 | R5  R5 | R5  R5 |
| VI-081 | CTRPSNNTRKSITIGPGQMFYRTGEIIGDIRKAYC | 76.1 | 35 | S/E | + | +3 | 52.8 | R5 | R5 |
| VI-086 | CTRPSNNTRTSITMGPGRVFYRTGEIVGDIRKAYC  ..........................T........ | 84.2  15.8 | 35  35 | S/E  S/E | +  + | +4  +4 | 5.7  6.3 | R5  R5 | R5  ND |
| VI-096 | CTRPSNNTRKSMTIGPGQVFYRTGDIIGDIRKAYC | 100.0 | 35 | S/D | + | +4 | 13.0 | R5 | R5 |
| VI-108 | CTRPSNNTRISMTVGPGQVFYRMGDIIGNIRKAYC | 100.0 | 35 | S/D | + | +4 | 5.3 | R5 | R5 |
| VI-120 | CTRPSNNTRTSIRIGPGQTFYRTGEIIGDIRKAYC | 100.0 | 35 | S/E | - | +4 | 29.7 | R5 | R5 |
| VI-213 | CTRPSNNTRRSMTIGPGQIFYRTGEIIGNIRKAYC | 100.0 | 35 | S/E | + | +5 | 24.0 | R5 | R5 |
| VI-219 | CTRPSNNTRTSITIGPGQVFYRTGEIIGDIRKAYC | 100.0 | 35 | S/E | + | +3 | 23.4 | R5 | R5 |
| VI-309 | CTRPSNNTRTSISMGPGRVYYRTGEVIGNIRQAFC | 100.0 | 35 | S/E | + | +3 | 4.0 | R5 | R5 |
| VI-310 | CTRPSNNTRISTRIGPGQVFYRTGEITGDIRKAYC | 100.0 | 35 | S/E | + | +4 | 1.9 | R5 | R5 |
| VI-313 | CTRPSNNIRTSTHMGPGRAFYRTGDIIGDIRKAYC | 100.0 | 35 | S/D | - | +4 | 1.7 | R5 | R5 |
| VI-404 | CTRPSNNTRTGIHIGPGQVFYRTGDIIGNIRKAYC  ....Y.....S.A....RM.....G...D...... | 99.9  0.1 | 35  35 | G/D  S/G | +  + | +4  +5 | 41.9  2.8 | R5  R5 | R5  ND |
| VI-410 | CTRPTGNTRESISFGPGRAFYRTGDIIGDIRKAYC  ....NN..I........QV..K..N.K......F. | 51.3  48.7 | 35  35 | S/D  S/N | -  + | +3  +3 | 21.2  6.1 | R5  R5 | R5  R5 |
| VI-421 | CTRPSNNTRTSVHMGPGQVFFRTGDITGDIRKAYC | 100.0 | 35 | S/D | + | +3 | 73.4 | R5 | R5 |
| VI-527 | CTRPYNNTRTSIAIGPGRMFYRTGGIIGDIRKAYC | 100.0 | 35 | S/G | + | +5 | 2.8 | R5 | R5 |
| VI-535 | CTRPNNNTRTSITIGPGQVFYRTGEIIGDIRKAYC | 100.0 | 35 | S/E | + | +3 | 19.1 | R5 | R5 |
| VI-031 | CTRPSTKVRISKRIGPGQVFYGTGAITGDRRTAYC  ........NI.....................K... | 64.8  35.2 | 35  35 | S/I  S/I | -  - | +5  +5 | 1.1  1.7 | X4/dual^i^  X4/dual | X4  X4 |
| VI-109 | CTRPSTNIRTSMTIGPGRVFYKTGAITGDIRKAYC | 100.0 | 35 | S/A | - | +4 | 1.3 | X4/dual | X4 |
| VI-185 | CTRP-YKTKTGVTRGLGRVFYRTGEVEGDIRKTYC | 100.0 | 34 | G/E | - | +5 | 0.5 | X4/dual | X4 |
| VI-301 | CTRP-YNTRTGITRGPGRVYYKTEEIVGDIRKAYC | 100.0 | 34 | G/E | - | +5 | 0.5 | X4/dual | X4 |

*^a^*Dots denote sequence identity. Dashes denote absence of amino acid. Only representative amino acid sequences are shown; *^b^*Amino acid residues at positions 11 and 25 of the V3 region; *^c^*PNGS, potential N-linked glycosylation site; *^d^*NC, net charge; *^e^*FPR, false-positive rate in Geno2Pheno_[coreceptor]_; *^f^*Genotype, coreceptor usage of the V3 region, as determined using the combined rule with 11/25, net charge, and PNGS; *^g^*Phenotype, coreceptor usage of each V3 region, as determined using pseudotype virus assay; *^h^*ND, not determined; ^i^X4/dual, CXCR4-using HIV-1 through genetic prediction.
