# Supplementary material for: Frequent Detection of HIV-1 Variants With Mixed Coreceptor Usage Among People Who Inject Drugs Infected With CRF01_AE: Possible Association With Coreceptor Switch
Source: Open Forum Infect Dis. 2026 Feb 21;13(2):ofag080. doi: 10.1093/ofid/ofag080 (PMC12951246; doi:10.1093/ofid/ofag080)
Supplement: ofag080_Supplementary_Data [file ofag080_supplementary_data.zip › Table_S4.docx]

Table S4. Relationship between tropism and plasma viral load and CD4 T cell counts

| Tropism  (phenotype) | Number of cases (%) | pVL, log_10_ copies/mL  [Median (IQR)*^b^*] | CD4, cells/μL  [Median (IQR)] |
| --- | --- | --- | --- |
| Pure R5 HIV-1 | 19 (53) | 4.84 (4.61-4.95) | 317 (105-419) |
| Pure X4/dual HIV-1 | 4 (11) | 4.41 (4.06-4.89) | 180 (101-339) |
| Pure total*^a^* | 23 (64) | 4.80 (4.45-4.94) | 279 (105- 408) |
| Mixed with R5 and X4/dual HIV-1 | 13 (36) | 5.15 (4.69-5.54) | 166 (78-287) |

*^a^*includes pure R5 and pure X4/dual HIV-1, Abbreviation: *^b^*IQR, interquartile range
