# Supplementary figures and images for: Frequent Detection of HIV-1 Variants With Mixed Coreceptor Usage Among People Who Inject Drugs Infected With CRF01_AE: Possible Association With Coreceptor Switch
Source: Open Forum Infect Dis. 2026 Feb 21;13(2):ofag080. doi: 10.1093/ofid/ofag080 (PMC12951246; doi:10.1093/ofid/ofag080)

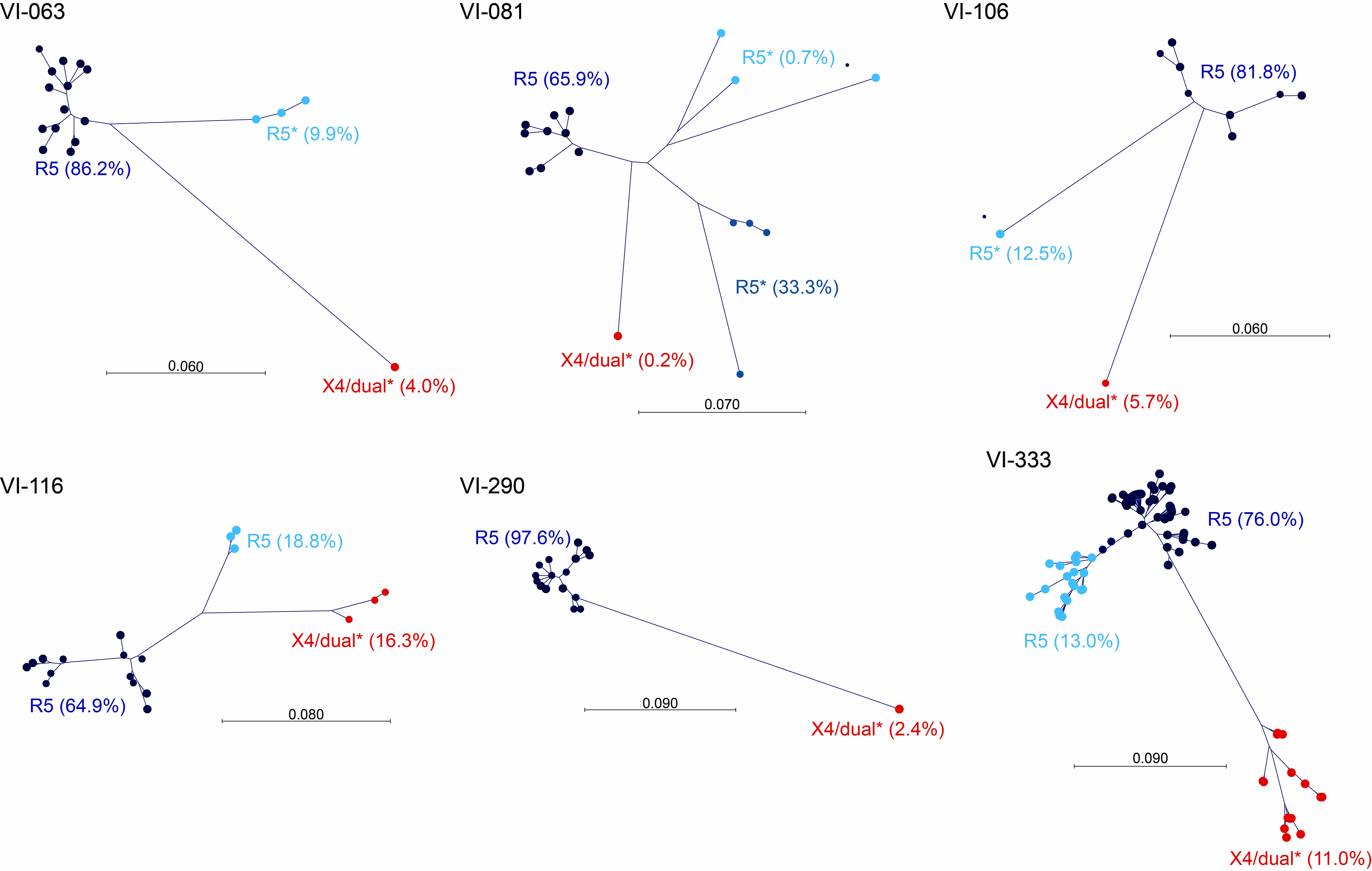

Supplement: ofag080_Supplementary_Data [file ofag080_supplementary_data.zip › Maeda_OFID_figure_S1.jpg]
